# Ageing, the Urban-Rural Gap and Disability Trends: 19 Years of Experience in China - 1987 to 2006

**DOI:** 10.1371/journal.pone.0012129

**Published:** 2010-08-13

**Authors:** Xiaoxia Peng, Shige Song, Sheena Sullivan, Jingjun Qiu, Wei Wang

**Affiliations:** 1 School of Public Health and Family Medicine, Capital Medical University, Beijing, China; 2 Institute of Sociology, Chinese Academy of Social Sciences, Beijing, China; 3 School of Exercise, Biomedical and Health Sciences, Edith Cowan University, Perth, Australia; 4 Graduate School, Chinese Academy of Sciences, Beijing, China; Yale University School of Medicine, United States of America

## Abstract

**Background:**

As the age of a population increases, so too does the rate of disability. In addition, disability is likely to be more common in rural compared with urban areas. The present study aimed to examine the influence of rapid population changes in terms of age and rural/urban residence on the prevalence of disability.

**Methods:**

Data from the 1987 and 2006 China Sampling Surveys on Disability were used to estimate the impacts of rapid ageing and the widening urban-rural gap on the prevalence of disability. Stratum specific rates of disability were estimated by 5-year age-group and type of residence. The decomposition of rates method was used to calculate the rate difference for each stratum between the two surveys.

**Results:**

The crude disability rate increased from 4.89% in 1987 to 6.39% in 2006, a 1.5% increase over the 19 year period. However, after the compositional effects from the overall rates of changing age-structure in 1987 and 2006 were eliminated by standardization, the disability rate in 1987 was 6.13%, which is higher than that in 2006 (5.91%). While in 1987 the excess due to rural residence compared with urban was <1.0%, this difference increased to >1.5% by 2006, suggesting a widening disparity by type of residence. When rates were decomposed, the bulk of the disability could be attributed to ageing, and very little to rural residence. However, a wider gap in prevalence between rural and urban areas could be observed in some age groups by 2006.

**Conclusion:**

The increasing number of elderly disabled persons in China and the widening discrepancy of disability prevalence between urban and rural areas may indicate that the most important priorities for disability prevention in China are to reinforce health promotion in older adults and improve health services in rural communities.

## Introduction

In China, persons aged 60 years and above, constituted 8.0% of the population in 1990 [1]; by 2007, this increased to 11.6% of the total population or 153.4million people [2]; and by 2050, the population aged 60 years and older will exceed 440 million and account for 31.1% of the total population [1], making China one of the most aged societies. Concomitant with this increase in the number of elderly, is likely to be an increase in the population with disabilities [3], [4].

Previous research has shown that age is strongly associated with disability. In Europe, 63% of people with disabilities are older than 45 [5]. This pattern is mainly due to deteriorating health that comes with age. A survey of disabled people in UK found that four out of five people reported some disability by age 80 years, of which cardiovascular disease and arthritis are the most common underlying causes [6]. Data from the European Community Household Panel (ECHP) [7], a large-scale representative survey, suggested that factors associated with the likelihood of reporting “non-hampered in daily activities by a chronic or mental health problem, illness or disability” included negative effects of ageing, unemployment and lower levels of education. A longitudinal, community-based study of older Australians followed for 104 months, also identified age as the strongest predictor of disability [8]. As the major force driving of the disability epidemic, there is little debate that the increasing population of elderly is becoming a major social issue.

Rural versus urban place of residence has also been shown to be a critical health determinant over time and across countries [9], [10]. A small number of recent studies from China have illustrated particularly wide urban/rural health inequalities among older adults, with a considerable urban advantage [11]. The study on disability in older adults based on the China National Sample Survey on Disability (CSSD) in 2006 demonstrated that rural residence was closely associated with lower socioeconomic status and significantly correlated with functional decline in older adults [12].

Other changes have taken place in China in the past two decades, which have resulted in changes in the prevalence of disability. First, the urban-rural income gap remains large and has increased somewhat over time [13]. Second, rural residents lack adequate access to healthcare, since the collectively-funded welfare programs (*hezuo yiliao*) were abandoned in most rural areas in the early 1980s and healthcare became predominantly employment-based. During these reforms the percentage of rural residents with some kind of health coverage was only 7.4%, which compared with 36.4% for urban residents [14]. By 2003, the gap in coverage between areas was still very wide, although coverage had improved somewhat to 12.6% and 49.6% in rural and urban areas, respectively [15]. Third, during the last two decades 100–200 million rural residents have migrated to urban areas for work, but most are given only short-term contracts which do not entitle them urban residency status, which in term precludes them from health care and other statutory benefits [16].

China has attempted to determine the prevalence of disability and rehabilitation needs of its country through two nationwide sampling surveys of disability (CSSD): one in 1987 and another in 2006. However, the urban-rural health divide has received limited attention to date in China despite its importance in the context of the country's development. The focus of this paper is to examine the possible trends of disability prevalence between two surveys and to explore the effects of both ageing and type of residence.

## Methods

Two CSSD surveys were national-representative household surveys conducted in 1987 and 2006. The first sampled a stratified sample of 369,816 households containing 1,579,314 persons (1.50% of the total Chinese population) [17]. The 2006 survey sampled 771,797 households and 2,526,145 persons (1.93% of the total Chinese population) [18]. Being household surveys they did not include institutionalized person, however questions were asked about all members officially resident within a sampled household.

In both surveys, four levels of sampling frame were used, including county or district of city (*shi*), town (*xiang*), village (*cun*) and community (*xiaoqu*). In the first step, the sampling frame was based on information on population, household, disability registration and economic status of counties which were collected by the provincial survey office according to the newest population and address information from the Ministry of Civil Affairs and Public Security. The target sample size for each level was determined according to the proportion of the provincial population resident at each level. In descending order, counties were sorted according to population size, accumulating the number of population by units and calculating the interval which was the total population divided by the number of units in each level. Then a number between 1 and the interval was selected as a random starting point for randomly sampling the counties. Finally, 734 counties accounting for 20% of all counties were sampled. Following the same method, four towns were sampled in each county, then two villages in each town and one community in each village. All persons in the sampled community were investigated. In sum, the survey sampled a total of 5,964 communities from 2,980 towns in 734 counties, with an average of 420 persons in each community. Results of post-survey quality checks showed that the omission rate of the resident population was 1.31/1000, the omission rate of the disabled population was 1.12/1000, and accuracy was greater than 95%. Thus, the sample was representative of the whole country and the data were reliable [12], [19]. Due to the multi-stage, stratified sampling used in the CSSD, it was necessary to use a weighting process to have prevalence estimates correctly reflect the Chinese population. In both surveys, every responder was assigned a weight corresponding to the number of people that respondent represented base on the Chinese Census.

Data were collected for five types of disability, namely physical, visual, hearing, and intellectual as well as mental health. According to the Law of the People's Republic of China on the Protection of Disabled Persons, a disabled person refers to one who suffers from abnormality in anatomical structure or loss of certain organ or function, psychologically, and who has lost wholly or in part the ability to perform an activity in the way considered normal. The impairment-based examination conducted in 1987 was also used in the 2006 survey. However, the International Classification of Functioning, Disability and Health (ICF) was considered in order to keep up with international developments in disability measurement in 2006, which led to two major changes: (1) disability was redefined, including mental disability and multiple-disability; (2) hearing disability that was combined with speech disability in 1987 survey was split into speech disability and hearing disability respectively in the 2006's survey. Thus, hearing disability has not been included in this analysis. The standards of visual, intellectual, mental and physical disability are available in Annex S1.

Data were obtained from the Office of The Second China National Sample Survey on Disability. Although individual-level data were obtained from the first survey, only stratified data were available for the 2006 survey. Thus, the data for the 1987 survey were first stratified by 5-year age-group and by type of residence (urban vs. rural) for each type of disability to match the summary data obtained for 2006. For the purpose of stratification, an urban population refers to the population residing in districts of a city, the population of “street committees” under the jurisdiction of a city, the population of “resident-committees” of townships under the jurisdiction of a city and the population of resident-committees of townships under the jurisdiction of a county. Chinese urban and rural distinctions are primarily the result of legal designations implemented after the establishment of the People's Republic of China in 1949, i.e. the household registration system (*Hu Kou*) divides the entire population. Rural residents who had migrated to urban areas for work were counted as rural residents in our study, although they were surveyed in sampled urban community if they lived there for longer than 1 year.

Within these strata, the prevalence rate of disability was estimated with 95% confidence intervals. The age-specific prevalence rate of disability was assessed by overlap of the 95% confidence intervals, where no overlap of confidence intervals suggests statistically different groups. As for the overall prevalence rate of disability, direct standardization was used to eliminate the compositional effects from the overall rates of changing age-structure between 1987 and 2006. The survey population was standardised to the population structure published in the 2010 World Population Prospects. The urban-rural gap of disability rate was tested by U-test. Data were managed and analyzed with SAS software version 9.1.3 (SAS Institute Inc, Cary, NC, USA, 2006).

The decomposition of the difference between two crude rates into several additive effects based on the purging method was developed by Liao [20], which integrates into a linear algebraic solution the rate standardization method based on a log-linear analysis. The modelling approach [21] (available at: https://netfiles.uiuc.edu/tfliao/www/decomposition/Decomposition.html) was used to explore the influence of rapid population changes in terms of age and rural/urban residence on the prevalence of disability. For example, if the cross-classificationinvolves two factors, namely age and residence, then the decomposition generates three additive effects: the age effect, the residence-effect and the rate-effect. Data were examined in terms of partial age-residence, partial age-residence and age-residence-year, marginal age-residence, and marginal age-residence and age-residence-year. The overall disability rate and prevalence rates of the four different types of disability, including visual, physical, intellectual and mental, were decomposed. For the sake of brevity, only the results from the marginal age-residence and age-residence-year analyses are reported here because there was little variation among the other results (which are available on request). Differences in the prevalence between years were compared by chi-square test with α = 0.05 for significance.

## Results

The data from the CSSD surveys indicated that the proportion of people aged 60 years and above increased from 8.88% in 1987 to 14.05% in 2006, while the proportion of people living in urban settings increased from 18.98% to 33.52% during the same time period. Table 1 shows the crude disability rates by 5-year age group, type of residence and year of data collection. As can be seen from the Table 1, the crude disability rate increased from 4.89% in 1987 to 6.39% in 2006, with a crude increase of 1.50% over the 19-year period. However, the standardized disability rate in 1987 was 6.13%, which is higher than that in 2006 (5.91%) after the compositional effects from the overall rates of changing age-structure in 1987 and 2006 were eliminated by standardization. In addition, the prevalence of disability among respondents aged 60 years and above was 21.93% and 24.03% in 1987 and 2006 respectively, higher than the overall prevalence of disability.

**Table 1:** Disability rates (%) for China by age and type of residence, 1987 and 2006.

Age	1987			2006		
	Total	Urban	Rural	Total	Urban	Rural
0–4	1.49 (1.43–1.55)	1.33 (1.19–1.47)	1.52 (1.45–1.59)	1.54 (1.47–1.61)	1.15 (1.04–1.26)	1.67 (1.59–1.75)
5–10*	2.85 (2.76–3.94)	2.27 (2.07–2.47)	2.96 (2.86–3.06)	1.65 (1.59–1.71)	1.15 (1.05–1.25)	1.86 (1.78–1.94)
10–14*	3.52 (3.43–3.61)	2.47 (2.27–2.67)	3.70 (3.60–3.80)	1.55 (1.49–1.61)	1.07 (0.98–1.16)	1.72 (1.65–1.79)
15–19*	2.33 (2.26–2.40)	1.96 (1.80–2.12)	2.40 (2.32–2.48)	1.78 (1.72–1.84)	1.38 (1.29–1.47)	1.93 (1.86–2.00)
20–24	2.25 (2.18–2.32)	1.62 (1.48–1.76)	2.39 (2.31–2.47)	2.21 (2.14–2.28)	1.50 (1.39–1.61)	2.57 (2.47–2.67)
25–29	2.51 (2.42–2.60)	2.05 (1.88–2.22)	2.67 (2.56–2.78)	2.48 (2.41–2.57)	1.61 (1.51–1.71)	3.01 (2.90–3.12)
30–34	2.88 (2.79–2.97)	2.03 (1.87–2.19)	3.12 (3.01–3.23)	2.99 (2.93–3.07)	1.94 (1.84–2.04)	3.63 (3.53–3.73)
35–39	3.64 (3.53–3.75)	3.11 (2.87–3.35)	3.77 (3.64–3.90)	3.48 (3.41–3.55)	2.46 (2.36–2.56)	4.04 (3.94–4.14)
40–44	4.27 (4.13–4.41)	3.52 (3.23–3.81)	4.45 (4.29–4.61)	4.18 (4.10–4.26)	3.20 (3.08–3.32)	4.76 (4.65–4.87)
45–49**	4.84 (4.68–5.00)	4.09 (3.78–4.40)	5.05 (4.87–5.23)	5.32 (5.21–5.34)	4.33 (4.18–4.48)	5.96 (5.82–6.10)
50–54**	6.02 (5.84–6.20)	4.76 (4.44–5.08)	6.41 (6.20–6.62)	6.38 (6.27–6.49)	4.97 (4.80–5.14)	7.12 (6.98–7.26)
55–59**	8.37 (8.15–8.59)	6.71 (6.30–7.12)	8.88 (8.62–9.14)	8.77 (8.62–8.92)	6.74 (6.51–6.97)	9.76 (9.57–9.95)
60–64	12.06 (11.77–12.35)	10.48 (9.90–11.06)	12.50 (12.17–12.83)	12.35 (12.15–12.55)	9.87 (9.55–10.19)	13.57 (13.32–13.82)
65–69	17.56 (17.17–17.95)	16.67 (15.84–17.50)	17.79 (17.36–18.22)	17.99 (17.74–18.24)	14.74 (14.35–15.13)	19.79 (19.46–20.12)
70–74	25.76 (25.24–26.28)	24.93 (23.77–26.09)	25.98 (25.39–26.57)	26.39 (26.07–26.71)	22.21 (21.71–22.71)	28.76 (28.35–29.17)
75–79	37.45 (36.69–38.21)	35.43 (33.71–37.15)	37.93 (37.08–38.78)	36.28 (35.85–36.71)	31.46 (30.76–32.16)	38.86 (38.32–39.40)
80–85*	49.29 (48.16–50.42)	45.97 (43.41–48.53)	50.08 (48.82–51.34)	46.66 (46.05–47.27)	41.76 (40.72–42.80)	49.15 (48.40–49.90)
85+*	58.86 (57.08–60.64)	56.61 (52.78–60.44)	59.49 (57.48–61.50)	55.90 (55.03–56.77)	52.68 (51.17–54.19)	57.52 (56.46–58.58)
Total	4.89 (4.86–4.92)	4.35 (4.28–4.42)	5.02 (4.98–5.06)	6.39 (6.36–6.42)	5.29 (5.24–5.34)	6.95 (6.91–6.99)

*The age-specific disability rate in 2006 is significantly (α = 0.05 for chi-square test) lower than that in 1987.

**The age-specific disability rate in 2006 is significantly (α = 0.05 for chi-square test) higher than that in 1987.

Age-specific disability rates in 2006 were significantly lower than in 1987 for both the youngest (aged from 5–19) and oldest (aged 80+ years) strata of the population. Conversely, the rates of disability were significantly higher in the age groups between 45 and 59 years. For the other ten age groups, the differences were not significant as their 95% confident intervals overlapped. However, rural residents did experience significantly increased rates of disability for all age groups between 20 and 75 years in contrary to the urban residents who had reduced rates for many of these age groups.

**Figure 1:**
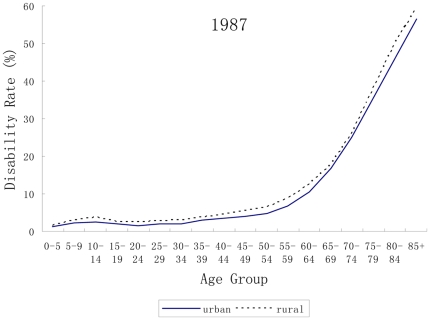
Disability rates by age and type of residence in 1987.

**Figure 2:**
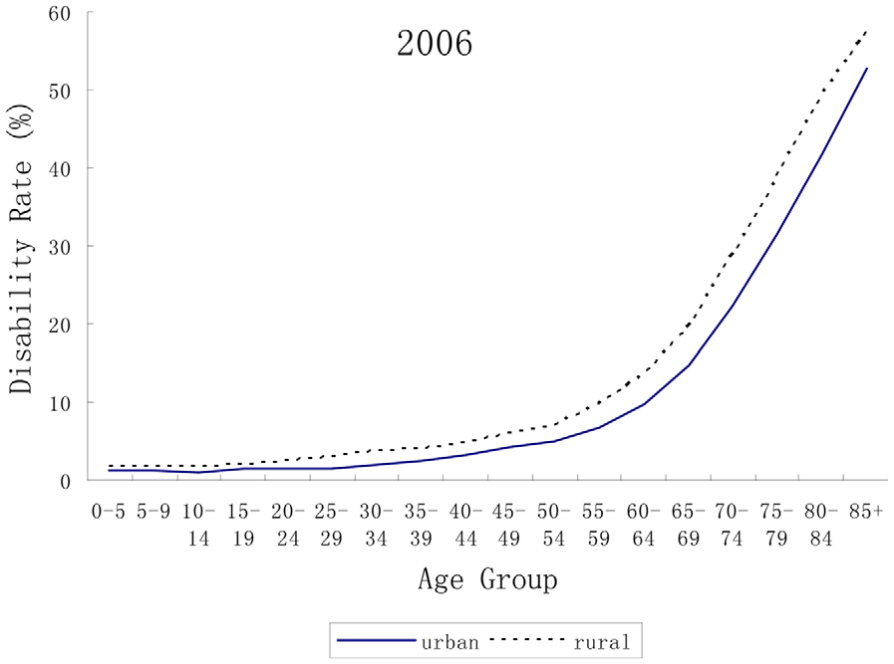
Disability rates by age and type of residence in 2006.

The disability rates by age group, type of residence in 1987 and 2006 are plotted in Figure 1 and Figure 2 respectively. It is clear that the gap in prevalence of disability between rural and urban areas increased from 1987 to 2006, particularly in the early-retirement years (60–75). Moreover, the overall prevalence of disability was consistently higher in rural areas than that in urban areas in both surveys (5.02% v.s. 4.35% in 1987; 6.95% v.s. 5.29% in 2006). The results of U-test indicated that the urban-rural gap was statistically significant (U = 45.00, p<0.0001).

Table 2 shows that the crude disability rate for 2006 was 1.50 points higher than that for 1987. However, if only the residence structure of the populations differed, as they did in the two survey years but the age structures and the age-residence-specific disability rates were identical in 1987 and 2006, then the overall disability rate in 2006 would be 0.02 points higher than that for 1987. The differences in the age and residence structures in 1987 and 2006 enhanced the difference between the crude disability rates in these two years. If the rates were standardized with respect to both age and residence, the difference between the standardized rates would be as low as 0.07.

**Table 2:** Component effects for the crude disability rates differences between 1987 and 2006.

Type	Crude rate difference	Component effects and percent distribution of effects (%)#			
		Age	Resident	Age*resident	Rate
Total	1.50	1.50	0.02	0.07	−0.09
	100.00%	100.00%	1.33%	4.67%	−6.00%
Visual	0.23	0.40	−0.01	0.01	−0.17
	100.00%	173.91%	−4.35%	4.35%	−73.91%
Physical	1.19	0.21	0.03	0.01	0.93
	100.00%	17.65%	2.52%	0.84%	78.15%
Intellectual	−0.54	−0.19	−0.01	−0.03	−0.31
	100.00%	35.18%	1.85%	5.56%	57.41%
Mental	0.28	0.07	0.03	0.00	0.18
	100.00%	25.00%	10.71%	0.00%	64.28%

# Percent distribution of effects: the crude rate difference divided by every component effect.

Furthermore, the percent distribution of effects indicated the possible bidirectional effects of ageing and urbanization on disability prevalence. So, the age-effect may be more than 100.00% when residence-effect is negative. The difference in prevalence of physical disability, intellectual disability and mental disability was expanded due to the effects of ageing and urbanization. The age-effect explained 17.65% of the increase in physical disabilities, 25% of the increase in mental disabilities, and 35.18% of the decline in intellectual disabilities. However, the difference in the prevalence of visual disability purged was the reverse of the crude difference because of impressive age-effect, explaining 173.91% of the increased rate of visual disabilities. By contrast, residence only explained 2.52% and 10.71% of the increases of physical and mental disability rates, and a decline of 4.35% and 1.85% of visual and intellectual disability rates, respectively.

## Discussion

The evidence was provided that a crude increase of 1.50% of disability rates from 1987 to 2006 was mainly due to that the dramatic ageing of population and the gap in prevalence of disability between rural and urban areas has widened, having important practical implications for China. As is consistent with previous reports [3], [4], [6], [12], our study shows that the disability prevalence increases with the advancement of age, especially after 60 years old. The prevalence of disability among Chinese elder aged 60 years and above is far higher than the overall prevalence of disability (21.93% vs. 4.89% in 1987; 24.03 vs. 6.39% in 2006). In fact, the disability prevalence of the elderly in some western countries (higher than 40.00%) is far higher than that in China due to the different measures of disability [22]–[24]. The measures of disability most widely used in western countries include the limitations in activities of daily living (ADLs) and instrumental activities of daily living (IADLs), whereas an impairment-based examination was used in China.

It is noticeable that the rates of disability between 1987 and 2006 increased for those in the 45–75 years old groups. This will translate to an enormous number of people who will need care in the future. Since we were not granted access to individual-level data for 2006 due to the certain regulations although we were one of the CSSD team members, we were unable to explore the effects of factors other than age and residence by multivariate statistical analysis. It is plausible that much of the increase seen in this study, particularly the increases seen in the older working population (those aged 45 to 59 years in 2006) in rural areas could be attributable to work injury [12]. The annual workplace fatality rate for 2001 was estimated at 11.1 per 100,000 workers in China, compared with a rate of 4.4 per 100,000 workers in the United States [25]. China's official records indicated that industrial accidents rose 27% from 2000 to 2001, and cases of occupational disease rose 13% in 2001 over 2000 [25]. Although substantial improvements in occupational health management have been made during the past two decades in China, this mainly has been happening in the big cities, such as Beijing and Shanghai. Therefore, China's occupational safety and health structure faces serious challenges in less developed areas, including a general lack of work safety awareness, poor and old infrastructure, and lax management. Under these conditions, the numbers of accidents, workplace injuries and occupational diseases have all increased, particularly in rural areas. One study found that 42% of farmers did not use personal protective equipment when working with pesticides, which was found to be strongly associated with the injury rate [26].

In contrast to the adult years, the rate of disability in younger age groups, 5–19 years, appeared to decline between the two surveys. This drop may be attributable to economic development, social progress and improvements in medical treatment and services. In the 1980s, morbidity of certain disabilities was effectively prevented through public health programmes in China, such as Healthy Birth and Sound Care, Planned Immunization, Iodine Supplementation and Intervention on Newborn Defects [18], [27].

The rural and urban difference is less salient in developed countries than in developing countries. Hence, few studies are available to examine the urban/rural residential relations with disability in developing countries [28]. In our study, one striking finding is the widening urban-rural discrepancy in disability prevalence over time although the decomposed rates indicated that the contribution of urbanization to overall disability was small in comparison with ageing. For those of working age and beyond, i.e. 20–75 years, the rates of disability among rural residents increased by more than two standard deviations between 1987 and 2006 in all age groups (Fig 1). However those living in urban areas enjoyed decreased rates of disability, except for the 45–60 years age groups. The potential explanations for the widening urban-rural gap in disability change in China are: (1) the over-sampling of disabled persons who are unable to migrate to urban areas for work may result in the over-estimation of disability rate in rural areas; (2) comparing with urban population aging, rural population aging is more serious, and the phenomenon of aged poverty is more extrusive in China [29]; (3) the harder life, poorer working standards, as well as limited availability of healthcare and rehabilitation services in rural areas may increase the risk of disability [26]. This discrepancy was coincident with the results of the Fourth National Health Services Survey in 2008, which found higher rates of disability among rural residents, especially among those aged 60+ years (33.8% vs. 26.0%) [30].

There are several limitations to this study. The disability classification and the criteria of disability identification were revised partly in 2006's survey to be in accordance with the International Classification of Functioning, Disability and Health (ICF) and the current situation of China. However, the disability measure based on impairment-based examination conducted in 2006 was similar to that in 1987, and thus the two surveys' data are comparable except for the data on hearing disability and speech disability. Another limitation was that we were unable to explore the effects of factors other than age and type of residence by multivariate statistical analysis in this study due to the limited access to individual-level data for 2006's survey. Nor could we disaggregate the rate differences between hearing and speech disability. In addition, underestimation of the crude disability prevalence in the youngest age group is likely because of possible underreporting by the parents who were not willing to report their children disability or who were not able to have definite clinical diagnoses of their young children at the time of the surveys [27].

Despite these limitations, the findings suggest that during the 19-year period from 1987 to 2006 China's society has become more disability-prone due to changes in age structure and a widening urban-rural gap. As for the causal analysis of the changing disability trends, further study is necessary. However, the burden of disability in China is only likely to worsen in coming years. Fortunately evidence suggests that a substantial proportion of chronic disabling conditions associated with aging are preventable, or at least able to be delayed, and that they are not an inevitable consequence when people are growing old [3], [12], [31]. Therefore, the most important priorities for disability prevention in China are to reinforce health promotion in older adults and improve health services in rural communities.

## Supporting Information

Annex S1 The standards of disability in CSSD.
(0.04 MB DOC)
