# Supplementary material for: Ageing, the Urban-Rural Gap and Disability Trends: 19 Years of Experience in China - 1987 to 2006
Source: PLoS One. 2010 Aug 13;5(8):e12129. doi: 10.1371/journal.pone.0012129 (PMC2921329; doi:10.1371/journal.pone.0012129)
Supplement: Annex S1 — The standards of disability in CSSD. (0.04 MB DOC) [file pone.0012129.s001.doc]

Annex: the standards of disability in CSSD

Table S1 Visual impairment can be divided into two categories, blind and low-vision

| Categories | Grading | standards |
| --- | --- | --- |
| Blind | 1 | single eyed vision is no greater than 0.02 or visual field is no less 5 degree |
|  | 2 | single eyed vision is no less than 0.05 and no greater 0.02 or visual field is no less 10 degree |
| Low-vision | 3 | single eyed vision is no less than 0.05 and no grater 0.1 |
|  | 4 | single eyed vision is no less than 0.3 and no greater than 0.1 |

Table S2 Intellectual impairment can be graded into four categories in accordance with the WHO and AAMD (American Association for Mental Retardation) standards

| Categories | DQ for children aged less than 6 years old | IQ for population aged more than 7 years old | Adaptation | Value of WHO-DAS* |
| --- | --- | --- | --- | --- |
| Very severely | ≤25 | ＜20 | very severely deficiency of adaptation | ≥116 |
| Severely | 26-39 | 20-34 | severely deficiency of adaptation | 106-115 |
| Moderate | 40-54 | 35-49 | moderate deficiency of adaptation | 96-105 |
| Less | 55-75 | 50-69 | mild deficiency of adaptation | 52-95 |

* **Mental Disability** was classified into four categories according the value of WHO-DAS.

Table S3 Physical handicap can be divided into four categories based on the parts and degrees of disability and functional barriers

| Categories | Grading | standards |
| --- | --- | --- |
| 1 | 0-2 | complete loss of ADL |
| 2 | 3-4 | basic loss of ADL |
| 3 | 5-6 | partial maintaining of ADL |
| 4 | 7-8 | basic maintaining of ADL |
